# Endotyping Seasonal Allergic Rhinitis in Children: A Cluster Analysis

**DOI:** 10.3389/fmed.2021.806911

**Published:** 2022-01-26

**Authors:** Velia Malizia, Giuliana Ferrante, Giovanna Cilluffo, Rosalia Gagliardo, Massimo Landi, Laura Montalbano, Salvatore Fasola, Mirella Profita, Amelia Licari, Gian Luigi Marseglia, Stefania La Grutta

**Affiliations:** ^1^Department of Biomedicine, Institute for Biomedical Research and Innovation, National Research Council, Palermo, Italy; ^2^Paediatric Unit, Department of Surgical Sciences, Dentistry, Gynaecology and Paediatrics, University of Verona, Verona, Italy; ^3^Pediatric National Healthcare System, Turin, Italy; ^4^Department of Pediatrics, Foundation IRCCS Policlinico San Matteo, University of Pavia, Pavia, Italy

**Keywords:** allergic rhinitis, children, cytokines, cluster analysis, endotypes

## Abstract

**Background:**

Seasonal Allergic Rhinitis (SAR) is a heterogeneous inflammatory disease. We hypothesized that a cluster analysis based on the evaluation of cytokines in nasal lavage (NL) could characterize distinctive SAR endotypes in children.

**Methods:**

This cross-sectional study enrolled 88 children with SAR. Detailed medical history was obtained by well-trained physicians. Quality of life and sleep quality were assessed through standardized questionnaires [Pediatric Rhinoconjunctivitis Quality of Life Questionnaire (PRQLQ) and Pittsburgh Sleep Quality Index (PSQI) respectively]. Children were grouped through K-means clustering using Interleukin (IL)-5, IL-17, IL-23, and Interferon (INF)-γ in NL.

**Results:**

Out of the 88 patients enrolled, 80 were included in the cluster analysis, which revealed three SAR endotypes. Cluster 1 showed lower levels of IL-5 and IL-17 and intermediate levels of IL-23 and IFN-γ; Cluster 2 had higher levels of IL-5 and intermediate levels of IL-17, IL-23, and IFN-γ; Cluster 3 showed higher levels of IL-17, IL-23, and IFN-γ and intermediate levels of IL-5. Cluster 1 showed intermediate values of nasal pH and nasal nitric oxide (nNO), and a lower percentage of neutrophils at nasal cytology than Clusters 2 and 3. Cluster 2 had a lower level of nasal pH, a higher nNO, higher scores in the ocular domain of PRQLQ, and worse sleep quality than Clusters 1 and 3. Cluster 3 showed a higher percentage of neutrophils at nasal cytology than Clusters 1 and 2.

**Conclusions:**

Our study identified three endotypes based on the evaluation of cytokines in NL, highlighting that childhood SAR is characterized by heterogeneous inflammatory cytokines.

## Introduction

The diversity of cytokines in nasal lavage (NL) fluid offers the opportunity of assessing the underpinning immune-inflammatory network of allergic rhinitis (AR) (1). Indeed, measuring such mediators in NL may contribute to describing the mucosal activity profile (2) and gaining insight into the pathophysiologic processes (3). A recent study on adult patients assessed nasal secretions, searching for potentially relevant mediators related to different rhinitis endotypes; however, despite a broad panel of inflammatory mediators, no clear profile could be found (4).

The pivotal role of Th2-derived cytokine polarization in seasonal AR (SAR) has been emphasized in a previous study in children, highlighting a close connection between Th2 cytokines, such as Interleukin-5 (IL-5), and eosinophil infiltration in the nasal mucosa (5). Interestingly, significantly increased levels of IL-17 were found in NL from adults with SAR, suggesting that the upregulation of Th17 may be involved in the inflammatory pathways of nasal disease in these patients (6). Moreover, the progressive decrease in the expression of IL-23p19 mRNA in response to specific allergen observed in the peripheral blood mononuclear cells of children with tree pollen-induced AR after sublingual immunotherapy suggests a role for this cytokine in the pathogenesis of SAR (7). Finally, low levels of IFN-γ have been described in NL from children with SAR, indicating that the Th1 immune response in the nasal mucosa is reduced in these patients (8). Overall, these data suggest that allergic inflammation is characterized by the activation of a complex immunological network, including a heterogeneous range of mediators. However, no study investigated whether different endotypes of childhood SAR could be characterized based on the cytokines that may be involved in the pathophysiological mechanisms underlying AR.

Data-driven approaches such as clustering methods could be useful for characterizing heterogeneous features of diseases among patients. In particular, the K-means algorithm is one of the most popular iterative descent clustering methods; it aims to minimize the sum of within-cluster variances and to maximize cluster separation, thereby identifying distinct groups within the population (9).

We hypothesized that a cluster analysis based on the evaluation of cytokines such as IL-5, IL-17, IL-23, and INF-γ in NL could characterize distinctive SAR endotypes in children.

## Materials and Methods

### Participants

We show the results of the first phase (to characterize the subjects at baseline and discriminate groups of children based on IL-5, IL-17, IL-23, and INF-γ in NL) of a cross-sectional study approved by the local Institutional Ethics Committee (Palermo 1, Italy, Approval Number: 10/2017). Once approved, the study was registered on ClinicalTrials.gov (NCT03349619). The study was conducted in accordance with Good Clinical Practice and the Declaration of Helsinki; informed consent was obtained from all parents before study entry.

The study population comprised 88 children with SAR. Children underwent a physical examination and were assessed for eligibility during their first consultation at the Institute for Biomedical Research and Innovation of the National Research Council (Palermo, Italy), between March 2018 and July 2018. The inclusion criteria were: (1) age 6–16 years; (2) diagnosis of AR in the previous year; (3) mono-sensitization to grass pollen, identified by positive skin prick test and specific immunoglobulin E (IgE >0.70 kU/l). The exclusion criteria were: (1) upper or lower respiratory tract infections (having taken antibacterial therapy in the 4 weeks before the study entry); (2) lifetime history of asthma (doctor diagnosis); (3) use of systemic/topical corticosteroids, systemic/topical decongestants, or antihistamines in the 4 weeks before the study entry; (4) anatomic nasal defects (i.e., septum deviation), or nasal polyps evaluated by a well-trained physician (VM) who performed a visual inspection through a headlamp with a nasal speculum in order to evaluate the anterior third of the nasal airway, including the anterior tip of the inferior turbinates and portions of the nasal septum; (5) active smoking. Pollen counts were monitored throughout the study period showing that grass pollen levels on average were below 30 grains/m^3^.

### Procedures

Detailed medical history was obtained by well-trained physicians (VM, GF, SLG) through a standardized questionnaire administered to parents (10–12) to investigate current AR symptoms and lifetime comorbidities, parental history of rhinitis, parental education, household crowding index (HCI, defined as the total number of co-residents per household, divided by the total number of rooms, excluding the kitchen and bathrooms), and information about exposure to current indoor (mold/pet/smoke) and outdoor risk factor (traffic at residential address). AR diagnosis was confirmed according to ARIA guidelines (13).

Current exposure to mold/pet/smoke at home was assessed through the questions: “Have you currently seen mold/dampness/fungi on the walls or on the ceiling of your child's bedroom?”; “In the past 12 months has your child had a pet (dogs or cats) at home?”; “Are there smokers at home”? Self-reported traffic exposure was recorded as the frequency of trucks passing on the street of residence on weekdays (never/rare/frequent/constant), and subjects were considered exposed if they answered “frequent” or “constant.”

### Nasal Parameters

The temporal sequence of nasal procedures was the following: nasal nitric oxide (nNO), nasal cytology and NL. All the procedures were performed on the same day within 30 min. Following ATS/ERS recommendations (14), nNO was measured ‘off line’ by an electrochemical sensor (Hypair FeNO, MediSoft, Belgium). Air from the nasal cavity was continuously analyzed by the sensor at a sample flow rate of 350 ml/min, during 30 s tidal breathing through resistance, so that the velum was closed to prevent any contamination of nasal with bronchial air. Only measurements with a variability <10% were retained. The mean nNO level was determined after three exhalations performed at >30-s intervals. nNO values were expressed as log nNO.

Nasal cytology was performed using a small plastic curette (Rhinoprobe TM) in anterior rhinoscopy, scraping from the middle portion of the inferior turbinate. The cellular material was spread on a glass slide, fixed by air drying, and then stained through the May-Grünwald-Giemsa method. Slides were read by a well-trained physician (ML) using a standard optical microscope equipped with a digital camera at ×1,000 magnification in oil immersion. The analysis of rhinocitograms involved the reading of not <50 fields. Granulocytes were assessed based on previously published recommendations (15).

NL was practiced by a well-trained physician (VM). Subjects were asked to tilt their head back at a 45° angle and close the nasopharynx with the soft palate. NL fluid was obtained by instilling 3 mL of isotonic saline (0.9% NaCl) prewarmed to 37°C in each patient's nostril, using a syringe. After 10 sec, the subject blew their nose forcefully into a sterile plastic container. The recovered NL vs. introduced volume saline solution was comparable. The average recovery of fluid from NL was approximately 70%. Obtained samples were transferred into conical polypropylene tubes and processed as previously described by Pizzichini et al. (16), with minor modifications (2). Briefly, dithiothreitol (DTT) (Sputolysin, Calbiochem Corp., San Diego, CA, USA), freshly prepared in a 10% dilution with distilled water, was added to the recovered NL fluid in the equivalent volume of 1/10th.

After homogenization and centrifugation at 500 g for 10 min at 4°C, the supernatant was stored at−80°C for later ELISA assay and pH measurements. Determinations of the absolute value of IL-5, IL-17, IL-23, and INF-γ in NL fluid were assessed using commercial ELISA kits, according to the manufacturer's instructions. The IL-5 sensitivity limit was 0.29 pg/ml (R&D Systems, Oxon, UK), IL-17 sensitivity limit was 0.01 pg/ml (Invitrogen, Thermo Fisher Scientific, Waltham, MA), IL-23 sensitivity limit was 4 pg/ml (Affimetrix, eBioscience, part of Thermo Fisher Scientific, Waltham, MA), IFN-γ sensitivity limit was 5 pg/ml (Abcam, Cambridge, MA). A stable pH was achieved in all cases after deaeration/decarbonation of the NL samples by bubbling with argon (350 ml/min) for 10 min. pH was measured in the sample using a pH meter (Corning 240, Science Products Division, New York, N.Y., USA) with a 0–14.00 pH Range 9. The pH meter was calibrated before each measurement using solutions with pH values of 4, 7, and 9.

All procedures were performed in a room maintained at a constant ambient temperature (23°C) and relative humidity (65%).

### Total 5 Symptom Score

The Total five Symptom Score (T5SS) is a subjective scoring system for the determination of symptom severity based on five domains. rhinorrhoea, nasal obstruction, nasal itching, sneezing, and eye itching. Each symptom is scored on a 4-point scale from 0 to 3 (0, absent; 1, mild – any symptom that is present but not particularly bothersome; 2, moderate – any symptom that is bothersome but does not interfere with daily activities or disturb sleep; 3, severe – any symptom that interferes with daily activity or disturbs sleep). The total score is calculated by adding the scores for all the five domains, resulting on a range of 0–15 (17).

### Pediatric Rhinoconjunctivitis Quality of Life Questionnaire

Quality of life was measured through the Italian validated version of the Pediatric Rhinoconjunctivitis Quality of Life Questionnaire (PRQLQ). PRQLQ is a self-administered disease-specific questionnaire, referring to the previous week, for assessing physical, emotional, and social problems in children with AR aged 6 to 12 years. It includes 23 items in five domains: nose symptoms, eye symptoms, practical problems, activity limitation, and other symptoms. Each domain is scored on a 7-point scale (from 0, not troubled, to 6, extremely troubled). The overall score is obtained as the mean score of all items and the domains score is the mean of the corresponding items (18).

### Pittsburgh Sleep Quality Index

The Pittsburgh Sleep Quality Index (PSQI) was used to assess sleep quality and disturbances. PSQI is a generic questionnaire completed by parents, with a 4-week recall, which includes 19 questions in 7 domains: subjective sleep quality, sleep latency, sleep duration, habitual sleep efficiency, sleep disturbance, use of sleep medications, and daytime dysfunction. Each domain is scored from 0 to 3. The overall score, ranging from 0 to 21, is obtained as the sum of each domain score. A total score above 5 indicates poor sleep quality (19).

### Statistical Analysis

Data were presented as n (%) or mean ±SD. Differences of categorical variables were analyzed using the Chi-squared test. Quantitative variables were compared using Kruskal Wallis test.

We applied K-means clustering (9) using standardized IL-5, IL-17, IL-23 and IFN-γ as the input variables. The optimal number of clusters was selected according to Elbow method (20), which runs k-means clustering on the dataset for a range of values for k (from 1 to 10 in our case) and plot a line chart of the total within sum of square for each value of k. If the line chart looks like an arm, then the “elbow” on the arm is the value of k that is the best. However, the determination of the number of clusters should also consider a combination of factors, including fit indices, cluster size, and interpretability (21). For cluster visualization, a “cluster plot” was drawn, plotting subjects in two dimensions after multidimensional scaling.

Between-cluster differences were tested by using the Kruskal-Wallis test. To check the robustness of our findings, a sensitivity analysis was performed imputing the missing values. Missing data were imputed using the mice (22) package in R, which creates multiple imputations (replacement values) for multivariate missing data. The Benjamini & Hochberg method was used to adjust the *p*-values for multiple comparisons. *P*-values lower than 0.05 were considered to indicate a statistically significant effect. Statistical analyses were performed through R version 3.6.2.

## Results

Demographic and clinical characteristics of the study sample are reported in Table 1. Out of 88 NL samples, 80 were includeed in the analysis (eight samples were below the detection limit of the ELISA kit).

**Table 1:** Characteristics of the study sample.

***n***	**88**
Age, years, median (IQR)	10.00 (4.00)
Range	6–16
Gender: Male	59 (67.05)
Height, cm	143.78 ± 16.39
Weight, kg	41.76 ± 16.64
BMI (kg/m^2^)	19.49 ± 4.51
Parental history of rhinitis (Y/N)	52 (59.09)
Parental education (>8 years)	69 (79.31)
Crowding Index	
0	1 (1.16)
1	42 (48.84)
≥2	43 (50.00)
Indoor exposure in the last 12 months^*^ (Y/N)	37 (42.53)
Outdoor exposure in the last 12 months^§^ (Y/N)	58 (66.67)
ARIA classes	
Mild Intermittent	20 (22.73)
Moderate-severe intermittent	28 (31.82)
Mild persistent	5 (5.68)
Moderate-severe persistent	35 (39.77)
Disease duration, years	3.94 ± 3.34
**Comorbidities** ^**∧**^	
Allergic conjunctivitis	15 (17.44)
Primary snoring	33 (37.93)
SPT wheal size (mm), median (range)^##^	4 (3–12)
**Nasal parameters**	
Log nNO, ppb	6.76 ± 1.10
Nasal cytology	
Log Eosinophils	−0.91 ± 3.08
Eosinophils	4.06 ± 6.18
Log Neutrophils	−2.47 ± 3.50
Neutrophils	7.26 ± 13.11
Log Mast cells	−2.64 ± 2.91
Mast cells	7.28 ± 0.23
Nasal pH^◦^	7.28 ± 0.23
IL-5 pg/ml^◦^	10.43 ± 11.48
IL-17 pg/ml^◦^	1.44 ± 2.50
IL-23 pg/ml^◦^	7.08 ± 4.08
IFN-γ pg/ml^◦^	6.49 ± 3.64
**Subjective measurements**	
Nasal symptoms	3.49 ± 1.52
Eye symptoms	2.51 ± 1.59
Practical problems	3.26 ± 1.63
Activity limitation	2.82 ± 1.38
Other symptoms	2.53 ± 1.33
***PRQLQ*** **total score**	2.92 ± 1.28
Sleep disturbance	2.33 ± 0.47
Sleep latency	0.82 ± 0.90
Day dysfunction due to sleepiness	0.49 ± 0.68
Sleep efficiency	1.00 ± 1.31
Self-rated sleep quality	0.94 ± 0.84
Need of medication to sleep	0.36 ± 0.94
***PSQI*** **total score**	5.95 ± 2.35
***T5SS***	8.56 ± 3.50
***T5SS** **≥6***	64 (80.00)

*Data are presented as n (%) or as mean ± SD.*

∧ *Lifetime;*

* *Exposure to mold/pets/smoke*.

§ *Exposure to traffic at residential address*.

## *Wheal and flare were measured, and saline-glycerin wheal size was subtracted from the grass wheal size*.

◦ *Missing data n = 8*.

*Y/N, Yes or No; BMI, Body Mass Index; ARIA, Allergic Rhinitis and its Impact on Asthma; SPT, Skin Prick Test; nNO, nasal Nitric Oxide; PRQLQ, Pediatric Rhinoconjunctivitis Quality of Life Questionnaire; PSQI, Pittsburgh Sleep Quality Index; T5SS, Total 5 Symptom Score*.

### Cluster Analysis

**Figure 1:**
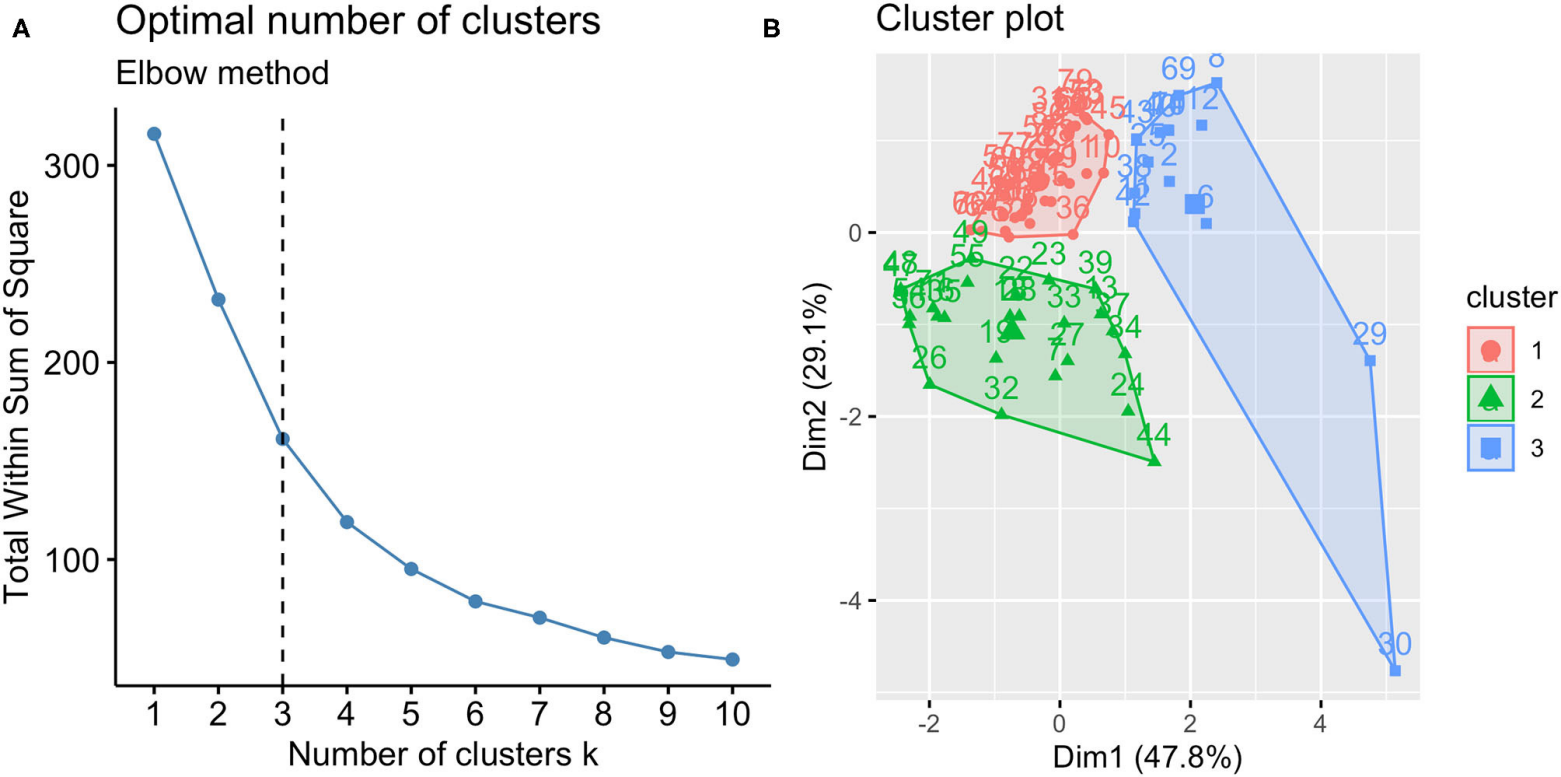
Optimal number of clusters **(A)** and cluster plot **(B)**. According to Elbow method, cluster size and interpretability the optimal number of clusters was 3; the clusters identified are reported in the cluster plot in which observations are represented by points, square and triangles using principal components.

**Figure 2:**
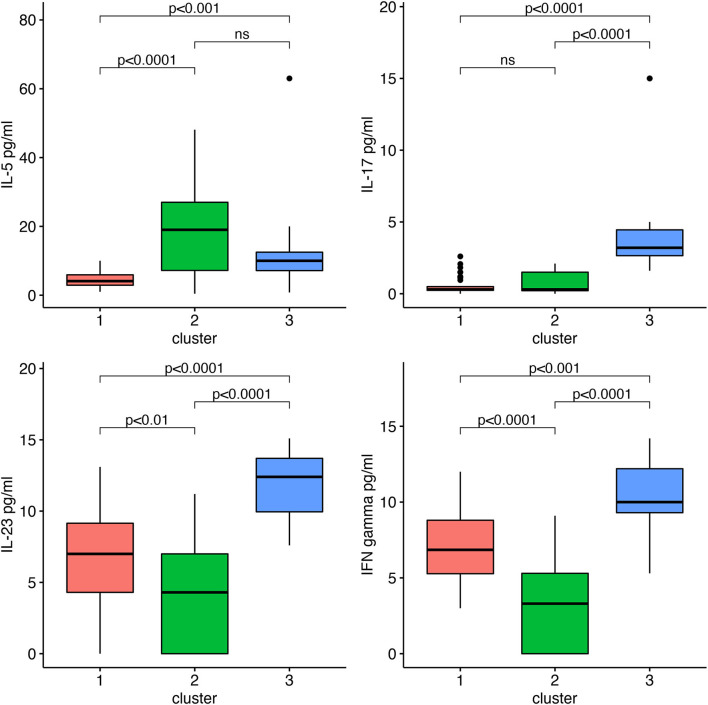
IL-5, IL-17, IL-23, and INF-γ by clusters.

According to the best trade-off between Elbow method, cluster size and interpretability, the optimal number of clusters was 3 (Figure 1A). Figure 1B highlights a good cluster separation. Figure 2 depicts IL-5, IL-17, IL-23 and INF-γ distribution in the three clusters.

**Cluster 1** comprised 40 (50%) children aged 9.78 ± 2.68 years; **Cluster 2** comprised 25 (31.2%) children aged 9.92 ± 2.31 years; and **Cluster 3** comprised 15 (18.8%) children, aged 10.47 ± 3.02 years. Multiple comparisons among clusters were reported in Table 2.

**Table 2:** Multiple comparisons among clusters.

	**Cluster 1**	**Cluster 2**	**Cluster 3**	**Cluster 1 vs Cluster 2**	**Cluster 1 vs Cluster 3**	**Cluster 2 vs Cluster 3**
	**40**	**25**	**15**	***p*-value**	***p*-value**	***p*-value**
**Nasal parameters**						
IL-5 pg/ml	4.40 ± 2.42	18.33 ± 12.92	13.33 ± 14.71	<0.001	<0.001	ns
IL-17 pg/ml	0.57 ± 0.60	0.79 ± 0.75	4.79 ± 4.25	ns	<0.001	<0.001
IL-23 pg/ml	6.97 ± 3.13	4.42 ± 3.71	11.82 ± 2.36	<0.001	<0.001	<0.001
IFN-γ pg/ml	7.05 ± 2.44	3.29 ± 3.09	10.41 ± 2.27	<0.001	<0.001	<0.001

### Cluster Characterization

Table 3 reports the clusters characterization. Cluster 1 showed intermediate values of nasal pH and nNO, and a lower percentage of neutrophils at nasal cytology than Clusters 2 and 3.

**Table 3:** Clusters characterization.

	**All**	**Cluster 1**	**Cluster 2**	**Cluster 3**	***p*-value**
***n***	**80**	**40**	**25**	**15**	
Age, years, median (IQR)	10.00 (4.00)	10.00 (3.25)	10.00 (3.00)	10.00 (5.00)	0.695
Gender: Male	53 (66.25)	27 (67.50)	14 (56.00)	12 (80.00)	0.291
Height, cm	143.93 ± 16.00	143.00 ± 16.49	145.40 ± 13.64	143.93 ± 19.02	0.779
Weight, kg	42.34 ± 16.80	43.33 ± 19.03	42.40 ± 14.19	39.60 ± 15.11	0.824
BMI, (kg/m^2^)	19.71 ± 4.54	20.27 ± 5.12	19.59 ± 4.06	18.42 ± 3.49	0.428
Parental history of rhinitis	50 (62.50)	24 (60.00)	16 (64.00)	10 (66.67)	0.886
Parental education (>8 years)	62 (78.48)	31 (77.50)	20 (80.00)	11 (78.57)	0.972
Crowding Index					0.841
0	1 (1.27)	1 (2.56)	0 (0.00)	0 (0.00)	
1	38 (48.10)	19 (48.72)	11 (44.00)	8 (53.33)	
≥2	40 (50.63)	19 (48.72)	14 (56.00)	7 (46.67)	
Current indoor exposure^#^ (Y/N)	34 (42.50)	17 (42.50)	12 (48.00)	5 (33.33)	0.662
Current outdoor exposure ^§^ (Y/N)	55 (68.75)	25 (62.50)	17 (68.00)	13 (86.67)	0.226
ARIA classes					0.211
Mild Intermittent	17 (21.25)	7 (17.50)	4 (16.00)	6 (40.00)	
Moderate-severe intermittent	27 (33.75)	16 (40.00)	6 (24.00)	5 (33.33)	
Mild persistent	5 (6.25)	1 (2.50)	3 (12.00)	1 (6.67)	
Moderate-severe persistent	31 (38.75)	16 (40.00)	12 (48.00)	3 (20.00)	
Disease duration, years	3.98 ± 3.33	4.00 ± 3.29	4.36 ± 3.32	3.27 ± 3.56	0.452
**Comorbidities** ^**∧**^					
Allergic conjunctivitis	14 (17.72)	6 (15.00)	5 (20.83)	3 (20.00)	0.812
Primary snoring	30 (37.50)	17 (42.50)	11 (44.00)	2 (13.33)	0.100
**Nasal parameters**					
Nasal cytology count					
Log Eosinophils	−1.09 ± 3.11	−1.52 ± 3.14	−1.22 ± 3.23	0.21 ± 2.63	0.282
Eosinophils	3.93 ± 6.39	3.54 ± 6.48	3.88 ± 6.44	5.00 ± 6.41	
Log Neutrophils	−2.35 ± 3.56	−2.96 ± 3.18	−2.65 ± 3.47	−0.37 ± 4.11	**0.043**
Neutrophils	7.74 ± 13.50	4.89 ± 10.50	6.96 ± 13.43	**16.00** **±17.36**	
Log Mast cells	−2.76 ± 2.87	−2.98 ± 2.75	−2.46 ± 3.15	−2.70 ± 2.83	0.764
Mast cells	1.95 ± 4.41	1.51 ± 3.53	3.00 ± 6.18	1.33 ± 2.58	
Nasal pH	7.28 ± 0.23	7.31 ± 0.19	7.17 ± 0.20	7.36 ± 0.32	**0.022**
Log nNO, ppb	6.86 ± 1.02	6.68 ± 1.12	7.35 ± 0.64	6.57 ± 1.01	**<0.001**
**Subjective measurements**					
Nasal symptoms	3.43 ± 1.48	3.34 ± 1.40	3.67 ± 1.66	3.25 ± 1.42	0.585
Eye symptoms	2.41 ± 1.54	2.29 ± 1.54	3.04 ± 1.73	1.68 ± 0.66	**0.029**
Practical problems	3.20 ± 1.57	3.05 ± 1.61	3.45 ± 1.56	3.17 ± 1.56	0.635
Activity limitation	2.53 ± 1.32	2.50 ± 1.31	2.95 ± 1.44	1.90 ± 0.91	0.055
Other symptoms	2.83 ± 1.40	2.86 ± 1.48	3.17 ± 1.36	2.21 ± 1.07	0.108
***PRQLQ*** **total score**	2.88 ± 1.26	2.81 ± 1.27	3.26 ± 1.35	2.44 ± 0.93	0.153
Sleep disturbance	2.31 ± 0.47	2.33 ± 0.47	2.36 ± 0.49	2.20 ± 0.41	0.560
Sleep latency	0.79 ± 0.88	0.80 ± 0.94	0.92 ± 0.91	0.53 ± 0.64	0.446
Day dysfunction due to sleepiness	0.49 ± 0.69	0.55 ± 0.75	0.56 ± 0.71	0.20 ± 0.41	0.211
Sleep efficiency	1.05 ± 1.33	0.82 ± 1.30	1.52 ± 1.33	0.87 ± 1.30	0.107
Self-rated sleep quality	0.95 ± 0.86	0.98 ± 0.86	1.04 ± 0.89	0.73 ± 0.80	0.475
Need of medication to sleep	0.34 ± 0.93	0.25 ± 0.81	0.44 ± 1.04	0.40 ± 1.06	0.768
***PSQI*** **total score**	5.94 ± 2.33	5.72 ± 1.77	6.88 ± 2.92	4.93 ± 2.15	**0.048**

*Data are presented as n (%) or as mean ± SD*.

∧ *Lifetime*.

# *Exposure to mold/pets/smoke*.

§ *Exposure to traffic at residential address*.

*Y/N, Yes or No; BMI, Body Mass Index; nNO, nasal Nitric Oxide; PRQLQ, Pediatric Rhinoconjunctivitis Quality of Life Questionnaire; PSQI, Pittsburgh Sleep Quality Index. p-values in bold were statistically significant*.

Cluster 2 had a lower level of nasal pH, a higher nNO, and higher scores of “eye symptoms” (as a domain of the PRQLQ), and PSQI total score, than Clusters 1 and 3.

Cluster 3 showed a higher percentage of neutrophils at nasal cytology than Clusters 1 and 2.

### Sensitivity Analysis

**Figure 3:**
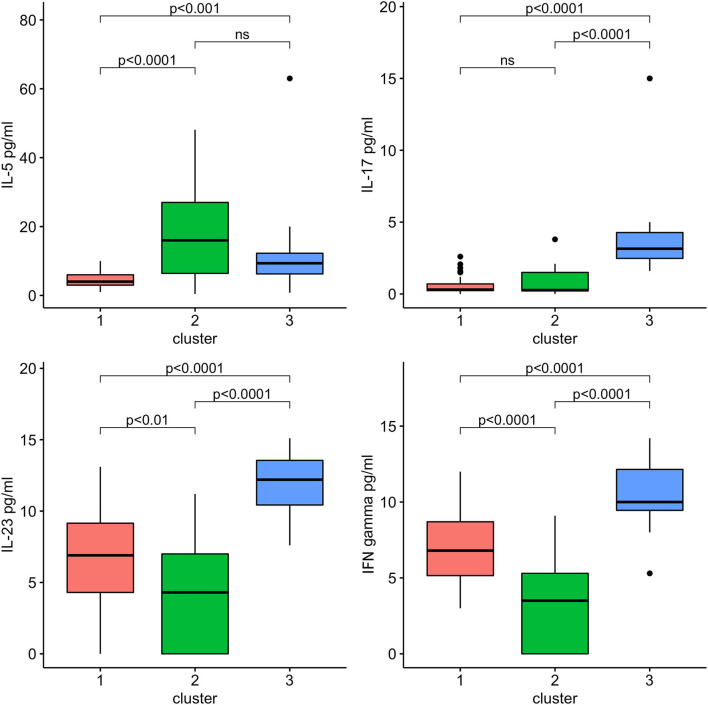
Sensitivity analysis: IL-5, IL-17, IL-23, and INF-γ by clusters.

After multiple imputations of missing values, the cluster analysis was re-run, and the new clusters were compared. Findings obtained with the new clustering did not change (Table 4 and Figure 3).

**Table 4:** Sensitivity analysis: clusters characterization after missing value imputation.

	**Cluster 1**	**Cluster 2**	**Cluster 3**	***p*-value**
***n***	**43**	**29**	**16**	
Age, years	10.00 (4.50)	10.00 (3.00)	10.50 (5.00)	0.634
Gender: Male	29 (67.44)	17 (58.62)	13 (81.25)	0.302
Weight, kg	42.93 ± 18.68	41.38 ± 14.71	39.31 ± 14.64	0.836
Height, cm	143.49 ± 17.29	144.10 ± 14.33	144.00 ± 18.37	0.952
BMI, (kg/m^2^)	20.01 ± 5.08	19.36 ± 4.14	18.31 ± 3.40	0.492
Parental history of rhinitis	25 (58.14)	18 (62.07)	10 (62.50)	0.926
Parental education (>8 years)	34 (79.07)	23 (79.31)	12 (75.00)	0.935
Crowding Index				0.751
0	1 (2.33)	0 (0.00)	0 (0.00)	
1	22 (51.16)	12 (41.38)	8 (50.00)	
≥2	20 (46.51)	17 (58.62)	8 (50.00)	
Current indoor exposure^#^ (Y/N)	19 (44.19)	13 (44.83)	5 (31.25)	0.626
Current outdoor exposure ^§^ (Y/N)	25 (58.14)	20 (68.97)	14 (87.50)	0.099
ARIA classes				0.104
Mild Intermittent	8 (18.60)	5 (17.24)	7 (43.75)	
Moderate-severe intermittent	17 (39.53)	6 (20.69)	5 (31.25)	
Mild persistent	1 (2.33)	3 (10.34)	1 (6.25)	
Moderate-severe persistent	17 (39.53)	15 (51.72)	3 (18.75)	
Disease duration, years	4.07 ± 3.40	4.17 ± 3.25	3.19 ± 3.45	0.474
**Comorbidities** ^**∧**^				
Allergic conjunctivitis	7 (16.28)	5 (17.24)	3 (18.75)	0.975
Primary snoring	17 (39.53)	13 (44.83)	3 (18.75)	0.208
**Nasal parameters**				
Nasal cytology				
Log Eosinophils	−1.28 ± 3.15	−1.04 ± 3.20	0.24 ± 2.54	0.437
Eosinophils	3.83 ± 6.35	3.96 ± 6.10	4.81 ± 6.24	
Log Neutrophils	−2.89 ± 3.23	−2.93 ± 3.28	−0.63 ± 4.11	0.054
Neutrophils	5.08 ± 10.51	5.96 ± 12.64	15.00 ± 17.24	
Log Mast cells	−2.66 ± 2.88	−2.52 ± 3.13	−2.81 ± 2.78	0.919
Mast cells	1.77 ± 3.63	2.93 ± 5.94	1.25 ± 2.52	
Nasal pH	7.31 ± 0.19	7.18 ± 0.20	7.38 ± 0.32	**0.014**
Log nNO, ppb	6.58 ± 1.21	7.06 ± 1.08	6.48 ± 1.05	**0.010**
**Subjective measurements**				
Nasal symptoms	3.47 ± 1.50	3.70 ± 1.61	3.14 ± 1.44	0.454
Eye symptoms	2.44 ± 1.70	3.06 ± 1.62	1.67 ± 0.64	**0.010**
Practical problems	3.16 ± 1.72	3.50 ± 1.56	3.08 ± 1.55	0.638
Activity limitation	2.83 ± 1.47	3.13 ± 1.32	2.20 ± 1.04	0.089
Other symptoms	2.46 ± 1.31	2.97 ± 1.43	1.94 ± 0.89	0.050
***PRQLQ*** **total score**	2.87 ± 1.33	3.27 ± 1.30	2.40 ± 0.91	0.091
Sleep disturbance	2.33 ± 0.47	2.41 ± 0.50	2.19 ± 0.40	0.306
Sleep latency	0.81 ± 0.93	1.00 ± 0.96	0.50 ± 0.63	0.239
Day dysfunction due to sleepiness	0.56 ± 0.73	0.55 ± 0.69	0.19 ± 0.40	0.140
Sleep efficiency	0.79 ± 1.30	1.34 ± 1.32	0.94 ± 1.29	0.182
Self-rated sleep quality	0.98 ± 0.86	1.00 ± 0.85	0.75 ± 0.77	0.543
Need of medication to sleep	0.26 ± 0.79	0.52 ± 1.09	0.38 ± 1.02	0.547
***PSQI*** **total score**	5.72 ± 1.78	6.86 ± 2.92	4.94 ± 2.08	**0.034**

*Data are presented as n (%) or as mean ± SD*.

∧ *Lifetime*.

# *Exposure to mold/pets/smoke*.

§ *Exposure to traffic at residential address*.

*BMI, Body Mass Index; nNO, nasal Nitric Oxide; PRQLQ, Pediatric Rhinoconjunctivitis Quality of Life Questionnaire; PSQI, Pittsburgh Sleep Quality Index. p-values in bold were statistically significant*.

## Discussion

To the best of our knowledge, this is the first study showing that SAR endotypes in children could be grouped into three distinct clusters based on the evaluation of cytokines in NL. Cluster 1 showed lower levels of IL-5 and IL-17 and intermediate levels of IL-23 and IFN-γ; Cluster 2 had higher levels of IL-5 and intermediate levels of IL-17, IL-23, and IFN-γ; Cluster 3 showed higher levels of IL-17, IL-23, and IFN-γ and intermediate levels of IL-5. Our findings highlight that childhood SAR is characterized by heterogeneous inflammatory cytokines. Previous studies on AR clusters have demonstrated the disease heterogeneity in adults and children (23–27), but only the study by Segboer et al. assessed nasal secretions for identifying different rhinitis endotypes in adult patients; however, despite a broad panel of inflammatory mediators, no clear profile could be found. In particular, differently from the current study, the authors failed to detect IL-5, IL-17, or IFN-γ in NL of pollen-sensitized AR patients (4). Such discrepancy could be ascribed to differences in study population that contribute to the variation observed between patients. In this regard, our results add further evidence attempting to define the NL cytokine profiling in children with SAR.

Non-invasive sampling of airway epithelial-lining-fluid by NL is a sensitive method to monitor inflammation in patients with respiratory diseases (28). The detection of inflammatory parameters in the nasal compartment may provide information about the general inflammatory status and might support the clinician in the framework of precision medicine targeted to pediatric allergic respiratory diseases (29, 30). Indeed, by measuring cytokines in NL, we were able to identify three distinct clusters of SAR in children.

### Cluster 1

Cluster 1 included 40 (50%) children, predominantly with moderate/severe intermittent and persistent severity level according to ARIA guidelines; this group showed intermediate values of nasal pH and nNO, and a lower percentage of neutrophils at nasal cytology than Clusters 2 and 3. Cluster 1 was characterized by a disease with a low overall inflammatory burden that did not correspond to a distinct Th1-, Th2-, or Th17-associated signature; therefore, it seems not associated with a specific polarized immune-inflammatory response.

### Cluster 2

Cluster 2 comprised 25 (31.2%) children, predominantly with moderate/severe persistent severity level according to ARIA guidelines; this group had a lower level of nasal pH, a higher nNO, and higher scores of “eye symptoms” (as a domain of the PRQLQ), and PSQI total score, than Clusters 1 and 3. Cluster 2 carried a predominant Th2 signature in the NL, given the significantly higher levels of IL-5 in this group than Clusters 1 and 3. IL-5 concentrations in both the upper and lower airways have been demonstrated to be related to the degree of eosinophilic inflammation in airway disease (31). Indeed, a previous study in children with SAR demonstrated the close connection between Th2 cytokines such as IL-5 and eosinophil infiltration in the nasal mucosa, emphasizing the pivotal role of Th2-derived cytokine polarization in SAR (5). It is accepted that the lower values of pH are associated with eosinophilic inflammation in the airways (31). Indeed, lower pH values were observed in the oral and nasal exhaled breath condensate (EBC) of children with asthma, atopic dermatitis, and AR with respect to healthy controls (29). Interestingly, the higher score of the domain “ocular symptoms” in PRQLQ as well as the higher PSQI total score highlight the higher burden of disease in this cluster in comparison with Clusters 1 and 3, which might be ascribed to a Th2 polarized immune-inflammatory response. These results are not surprising, as we previously demonstrated that QoL and sleep quality may be impaired in children with perennial and seasonal AR (32, 33). In particular, ocular allergic symptoms are strongly associated with AR and are increasingly recognized as a disorder that imposes its burden on patient's QoL (34). A significant association between AR and poor sleep quality has also been reported (35). The finding of higher nNO levels in Cluster 2 is in line with a predominant Th2 signature in this group of patients. Indeed, allergen exposure and consequent inflammation in the nose and paranasal sinuses lead to mast cells' activation and antigen-specific Th2 cells with the simultaneous production of cytokines, including IL-5 (36). In patients with AR, nNO may be used as a biomarker of eosinophilic inflammation because nasal eosinophils displayed the best correlation with symptoms and inflammation in AR (37). In addition, the levels of nNO positively correlated with the levels of IL-5 in nasal EBC of children with AR, as we previously demonstrated (29). Nonetheless, we could not observe a significantly different percentage of eosinophils at nasal cytology. We can hypothesize that a low accumulation of eosinophils in the nasal mucosa of our children could be due to a low extent of the epithelial damage which in turn could be ascribed to the low (below 30 grains/m^3^) grass pollen levels monitored throughout the study period.

### Cluster 3

Cluster 3 included 15 (18.8%) children, mainly with mild intermittent severity level according to ARIA guidelines, and carried a predominant Th1/Th17 signature in the NL, as suggested by the significantly higher levels of IL-17, IL-23, and IFN-γ in this group than Clusters 1 and 2. The IL-23/IL-17 pathway is related to the local tissue inflammatory response. It is thought that IL-23 plays a significant role in the early stages of allergic inflammatory responses, as it directly induces IL-17 production and neutrophil migration and accumulation (38). Indeed, IL-23 is significant in the antigen-dependent activation of both Th2 and Th17 cells as well as in the active phase of allergic respiratory tract inflammation (39, 40). Here, the significance of IL-17 and IL-23 in Cluster 3 and the relevant increase of nasal neutrophils in NL suggest a potential regulatory role of IL-23–Th17 axis in nasal inflammation in this group of children. The concomitant overexpression of IFN-γ, IL-23, and IL-17 in Cluster 3 suggests a potential involvement of IFN-γ in reducing Th2 profile and an endotypic switch toward IL-23/Th17 axis in this group of children with SAR. Indeed, IFN-γ acts not only as a potent activator of the Th1 phenotype but also as a suppressor of Th2 development (41). The suppressive effects of IFN-γ on allergic diseases have been shown to be mediated by various mechanisms, such as the regulation of allergen presentation to T lymphocytes, the differentiation of naive T cells toward Th1 phenotype and/or inhibition of Th2 cell recruitment/differentiation, the suppression of Th2 cytokine release from activated T cells, and the induction of apoptosis in T cells and eosinophils (42–45).

Overall, the inflammatory endotypes outlined in this study confirm that SAR is a heterogeneous inflammatory disease and suggest that clustering methods could be useful for characterizing heterogeneous features of AR within distinct patients.

### Strengths and Limitations

The main strength of our study is the application of an unsupervised statistical method, i.e. cluster analysis, to a population of subjects with a wide range of nasal parameters to identify the possible underlying endotypes. With respect to hierarchical clustering, K-means is computationally faster, relatively scalable, and simple, and can produce tighter clusters. Moreover, clinical characterization followed the international ARIA classification and standardized questionnaires were used to evaluate the disease burden.

We also recognize some study limitations. First, we did not test peripheral specific and total IgE as markers of systemic allergic inflammation; however, comparable levels of eosinophil inflammation in the blood and airways have been previously reported in patients with AR regardless of the pollen season (46). Moreover, we could not perform the cluster analysis on the overall study sample, given that eight samples were considered non-viable due to little or no protein content upon analysis; however, after sensitivity analysis, results did not change. No other Th2 cytokines as for example levels of IL-4 and IL-13 in nasal secretions were measured. A control group could be considered in further studies in order to have a normal reference group. To our knowledge there is no evidence that rhinoprobe scraping may alter the wash content, however, testing for confirm this hypothesis would have been useful. Finally, our subjects were cross-sectionally analyzed; therefore, validation of the three clusters identified in this study will be necessary to assess whether the results obtained are maintained, and especially with higher pollen exposure. Moreover, more extensive longitudinal studies are required, for assessing cluster stability over time, also in relation to treatment and environmental exposures, and generalizability of our findings to other populations.

## Conclusions

In conclusion, this is the first study reporting different inflammatory SAR endotypes in a pediatric population based on K-means clustering method, and the first one using a minimally invasive collection of nasal cytokines for this purpose. Endotypes may provide a more accurate description of the inflammatory patterns than phenotypes only; in this regard, SAR-related inflammation in children should be considered multi-dimensionally heterogeneous on the Th1, Th2, Th17 axes. The clinical implications of the current findings would benefit from future studies that are required to assess the stability of inflammatory signatures over time.

## Data Availability Statement

The raw data supporting the conclusions of this article will be made available by the authors, without undue reservation.

## Ethics Statement

The studies involving human participants were reviewed and approved by EC Palermo 1, Italy, Approval Number: 10/2017. Written informed consent to participate in this study was provided by the participants' legal guardian/next of kin.

## Author Contributions

SLG designed the study. GC contributed to data analysis, interpretation, and to draft of the article. SLG, GF, and AL wrote the initial draft. SF and GLM contributed to the interpretation of the results and reviewed the manuscript. VM, ML, RG, MP, and LM mainly contributed to data collection. All authors actively participated in all phases, and agreed to be accountable for the accuracy and integrity of any part of the work.

## Conflict of Interest

The authors declare that the research was conducted in the absence of any commercial or financial relationships that could be construed as a potential conflict of interest. The reviewer RC declared past collaborations with the authors AL and GLM to the handling editor.

## Publisher's Note

All claims expressed in this article are solely those of the authors and do not necessarily represent those of their affiliated organizations, or those of the publisher, the editors and the reviewers. Any product that may be evaluated in this article, or claim that may be made by its manufacturer, is not guaranteed or endorsed by the publisher.
